# Structure-Function Insights into the Dual Role in Nucleobase and Nicotinamide Metabolism and a Possible Use in Cancer Gene Therapy of the URH1p Riboside Hydrolase

**DOI:** 10.3390/ijms25137032

**Published:** 2024-06-27

**Authors:** Alejandra Angela Carriles, Laura Muzzolini, Claudia Minici, Paola Tornaghi, Marco Patrone, Massimo Degano

**Affiliations:** 1 Biocrystallography Group, IRCCS San Raffaele Scientific Institute, Via Olgettina 60, 20132 Milano, Italy; 2 Faculty of Medicine and Surgery, Università Vita-Salute San Raffaele, Via Olgettina 58, 20132 Milano, Italy

**Keywords:** NAD^+^, nucleosides, metabolism, enzymology, X-ray crystallography, cancer therapy

## Abstract

The URH1p enzyme from the yeast *Saccharomyces cerevisiae* has gained significant interest due to its role in nitrogenous base metabolism, particularly involving uracil and nicotinamide salvage. Indeed, URH1p was initially classified as a nucleoside hydrolase (NH) with a pronounced preference for uridine substrate but was later shown to also participate in a Preiss-Handler-dependent pathway for recycling of both endogenous and exogenous nicotinamide riboside (NR) towards NAD+ synthesis. Here, we present the detailed enzymatic and structural characterisation of the yeast URH1p enzyme, a member of the group I NH family of enzymes. We show that the URH1p has similar catalytic efficiencies for hydrolysis of NR and uridine, advocating a dual role of the enzyme in both NAD^+^ synthesis and nucleobase salvage. We demonstrate that URH1p has a monomeric structure that is unprecedented for members of the NH homology group I, showing that oligomerisation is not strictly required for the N-ribosidic activity in this family of enzymes. The size, thermal stability and activity of URH1p towards the synthetic substrate 5-fluoruridine, a riboside precursor of the antitumoral drug 5-fluorouracil, make the enzyme an attractive tool to be employed in gene-directed enzyme-prodrug activation therapy against solid tumours.

## 1. Introduction

NAD^+^ (Nicotinamide Adenine Dinucleotide) metabolism stands as a crucial cornerstone in the intricate network of cellular processes. Operating as a coenzyme, NAD^+^ participates in a multitude of fundamental biological reactions, ranging from energy production to DNA repair and cellular signalling [1]. Its dual role both as a cofactor for redox reactions and as a regulator of diverse metabolic processes underscores its significance in maintaining cellular health and functionality. Indeed, NAD^+^ metabolism intersects with vital pathways such as glycolysis, the citric acid cycle, and oxidative phosphorylation, fostering an essential nexus with energy production. Furthermore, NAD^+^ exerts regulatory functions by modulating gene expression and influencing processes like ageing and stress response through the activities of enzymes such as sirtuins [2] and ADP-ribosyltransferases [3]. Given its extensive impact, deciphering the nuances of NAD^+^ metabolism may not only enrich our understanding of basic cellular physiology but also unveil potential therapeutic avenues for a range of diseases, including metabolic disorders [4], neurodegenerative conditions [5], infectious diseases [6,7] and cancer [8].

While energetic metabolism does not require a steady synthetic flow of the cofactor, several NAD-consuming enzymes, such as ADP-ribosyltransferases [9] and poly-ADP-ribose polymerases [10], decrease the cellular concentration of NAD^+^. Thus, adequate levels of NAD^+^ are maintained through the intricate interplay of chemical reactions balancing, on the one hand, its physiological breakdown and, on the other, its de novo synthesis and salvage. Most bacteria and eukaryotes can synthesise the pyridine base starting from the amino acids aspartate or tryptophan, while some parasitic organisms are apparently NAD^+^-auxotrophic and rely on a conserved network of proteins devoted to the uptake and salvage of the nicotinamide ring [11].

**Scheme 1:**
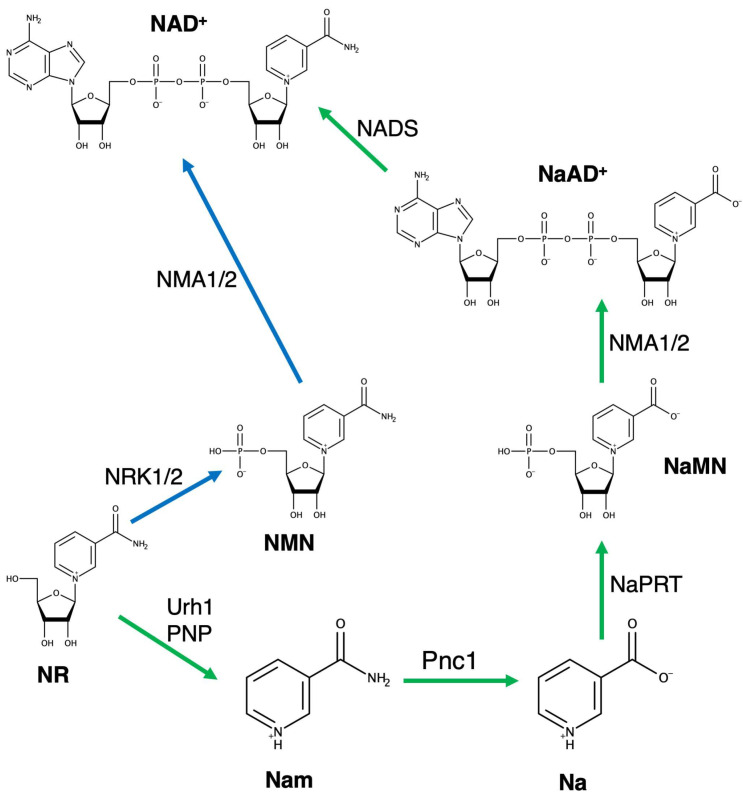
Metabolic pathways in eukaryotes involved in the conversion of nicotinamide riboside (NR) to NAD^+^. Blue arrows indicate the NR kinase (NRK1/2) dependent pathway and green arrows indicate the URH1/PNP-mediated conversion. NMA1/2 nicotinamide mononucleotide adenyltransferase; Pnc1, nicotinamidase; NaPRT, nicotinate phosphoribosyltransferase; NADS; NAD^+^ synthetase.

In recent years, the intermediate metabolite nicotinamide riboside (NR) [12] has been demonstrated as a central player in maintaining NAD^+^ levels in *Saccharomyces cerevisiae*. Indeed, NR enhances the sirtuin Sir2-dependent repression of recombination and telomeric silencing and extends the average lifespan of *S. cerevisiae* NRK1 mutants [13]. Two main enzymatic activities play a pivotal role in the metabolism of NR towards NAD^+^ synthesis [12] (Scheme 1). The first takes advantage of two ATP-dependent NR kinases, NRK1 and 2, catalysing the direct phosphorylation to nicotinamide mononucleotide. The second pathway (Preiss-Handler) converts the free nicotinamide base to nicotinic acid via nicotinamidase and then to the nicotinic acid mononucleotide via a nicotinate phosphoribosyltransferase-catalysed reaction. The conversion of NR to nicotinamide (Nam) is catalysed by nucleoside phosphorylases in vertebrates [14]. In other organisms, enzymes with nucleoside/riboside hydrolase (NH) activity also catalyse the N-ribosidic bond cleavage, yielding Nam as a NAD^+^ precursor [11]. In yeast, the concerted enzymatic action of both the URH1p NH and the nucleoside phosphorylase PNP1 represent a crucial entry point for exogenous NR into the NAD^+^ salvage pathway [15]. This pathway is also active in the absence of added NR and allows cells to recycle and regenerate NAD^+^ molecules.

The discovery of their role in the hydrolytic conversion of NR to Nam expanded the physiological role of NH enzymes. NHs were originally identified as nucleoside hydrolases in purine-auxotrophic parasitic protozoa [16] that lack nucleoside phosphorylase activity and, thus, apparently employ the N-glycosidic bond hydrolysis of nucleosides to recycle purine bases for nucleotide synthesis. Further work demonstrated that NHs are widespread throughout evolution, albeit homologous genes are not found in mammals [17,18]. NH enzymes can have diverse substrate preferences, and purine-specific [19,20,21], pyrimidine-specific [18,22,23,24] and non-specific isozymes [25,26,27] have been described. The demonstration that other naturally-occurring N-ribosides such as NR [11,15], modified nucleosides such as 5-fluorouridine (FUR) [11,18,28], as well as O-ribosides [17,29] are substrates of NH proteins suggests that these enzymes may be better described as ribosyl hydrolases, and most importantly participate in diverse metabolic processes [30]. The yeast URH1p [31,32], a cytosolic enzyme, was found to exhibit uridine and NR hydrolase activity [15], contributing both to the cell’s ability to recover uracil for nucleotide biosynthesis and maintain a balanced pool of nucleotides for DNA, RNA and cofactor synthesis and to regulate NAD^+^ concentration through the Preiss-Handler shunt. Indeed, in yeast cells, URH1p is expressed at higher levels than PNP1 and shows a higher catalytic efficiency towards NR compared to phosphorylase [15]. Thus, URH1p encodes a pivotal activity for the NAD^+^ homeostasis in *S. cerevisiae*.

Aside from the investigation of their physiological role, NHs have also been proposed as a potential tool for gene-directed enzyme prodrug activation therapy (GDEPT) against cancer [18,33,34]. Indeed, purine-specific NHs have been used to activate 6-methylguanosine to the corresponding cytotoxic base and successfully delivered to cancer cells. Another strategy can involve the ectopic expression of pyrimidine-preferring NHs to promote the localised conversion of the lowly toxic and more bioavailable prodrug 5-fluorouridine (FUR) to the 5-fluorouracil base. The subsequent formation of the 5-fluorouridylate molecule leads to several cytotoxic events, including the synthesis of faulty RNA molecules and the covalent inhibition of thymidylate synthase, depriving cells of deoxythymidine nucleotides. The tight junction-mediated transfer of toxic metabolites leads to a beneficial bystander effect. In mammalian cells, the conversion of FUR to the nucleobase is a poorly efficient process catalysed by purine nucleoside phosphorylases. Thus, the identification of enzymes with high catalytic efficiency towards FUR and adequate thermal stability is an essential step towards the development of such an approach.

Here, we present the full biochemical, enzymatic and structural characterisation of the URH1p enzyme from *S. cerevisiae*. We show that URH1p catalyses the hydrolysis of NR and uridine with similar catalytic efficiencies, thus suggesting that both these ribosides are processed by the enzyme under physiological conditions. The URH1p-catalyzed hydrolysis of NR at 37 °C does not follow simple Michaelis-Menten kinetics and is modulated by substrate inhibition. The crystal structure of URH1p showed that the enzyme belongs to the group I structural homology group of the Ca^2+^-dependent NHs and displays a monomeric structure that is unprecedented in this class of enzymes. The structure, thermal stability, and efficient catalysis of FUR hydrolysis make URH1p an interesting candidate for a GDEPT approach against solid tumours.

## 2. Results

### 2.1. The URH1p Protein Is a Member of the NH Structural Homology Group I

The *urh1* gene from *S. cerevisiae* encodes for a 340-amino acid protein (URH1p, Uniprot entry Q04179) with a molar mass of 27,960 Da and a predicted isoelectric point of 5.2 that contains an aspartate-rich fingerprint N-terminal sequence (DXDXXXDD) that is considered diagnostic for NH-like proteins [30]. The gene has two possible translation initiation sites, and previous work showed that the long and short isoforms are equally able to rescue the phenotype of a deletion mutant [28]. URH1p contains two histidine residues (His86 and His254) that are conserved in the structural homology group I of NHs [18], a group of enzymes acting on either both purine and pyrimidine nucleosides (non-specific, termed IU-NHs for inosine-uridine-preferring) or pyrimidine-preferring (cytidine-uridine or CU-NHs) [35].

**Figure 1:**
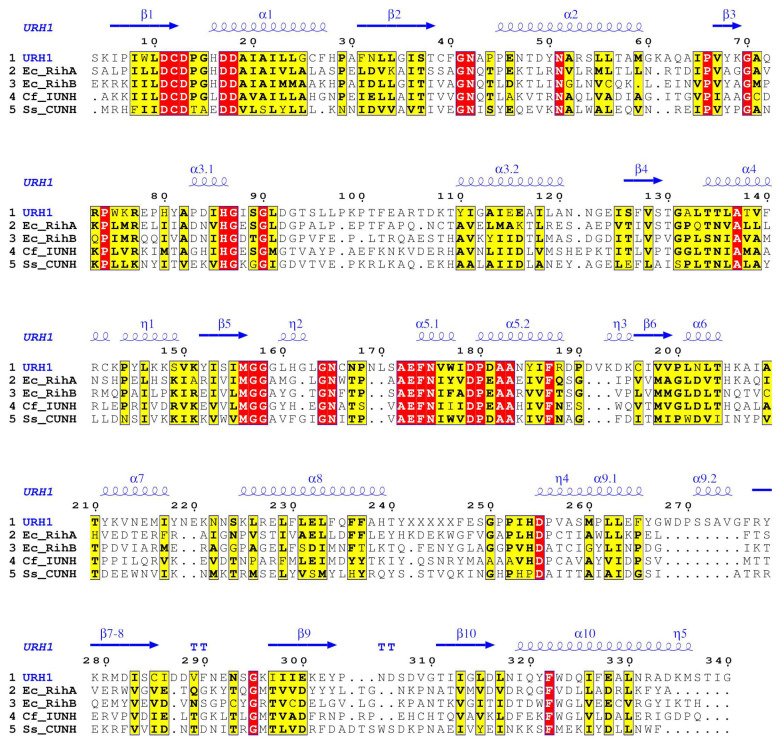
Alignment of the amino acid sequence of URH1p with representative members of the NH family. Secondary structure elements in the first line are derived from the URH1p crystal structure, here determined. Regions with strictly conserved amino acids are highlighted in red and similar ones in yellow and bold typeface. RihA and RihB are pyrimidine-selective NHs from *E. coli*, IU-NH is the non-specific NH from *C. fasciculata*, CUNH is the pyrimidine-specific NH from *S. solfataricus*.

Mutagenesis and molecular dynamics calculations have implied these histidine residues in the catalytic mechanism of these enzymes [17,18,36]. Moreover, as previously noted, URH1p contains only one of the two hydroxylated residues at the α8 helix that are crucial to catalyse the hydrolysis of purine nucleoside substrates [35]. Members of bacterial and parasitic group I NH are characterised by a tetrameric quaternary structure, while the corresponding isozymes from plants so far characterised are primarily dimeric [37]. URH1p is longer in amino acid sequence compared to most bacterial and trypanosomal NHs, typically ranging between 310 and 315 amino acids. Multiple sequence alignment (Figure 1) with representative homologues indicates that the additional amino acids are inserted at the long loop connecting strand β5 to helix α5.1, between helix α5.2 and strand β6, and at the N-terminus of strand β8. All these regions are remote from the active site, thus unlikely to have substantial effects on the enzymatic activity, rather affecting the regions classically involved in group I NH oligomerisation [30].

**Figure 2:**
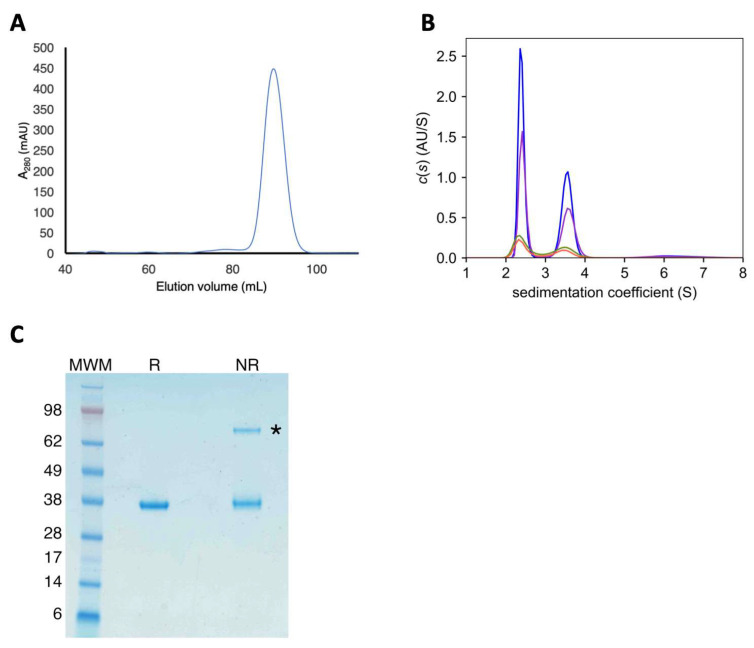
Biochemical analysis of the URH1p protein. (**A**) Size exclusion chromatography of the freshly purified protein shows a single, monomeric species. (**B**) Sedimentation velocity AUC of aged samples shows the presence of a dimeric species at 3.7 S. Curves correspond to protein concentrations of 2.6 (red), 5.2 (green), 10.4 (purple) and 15.6 µM (blue), respectively. (**C**) Disulfide-linked URH1p dimers are apparent in SDS PAGE analysis of non-reduced samples, highlighted by an asterisk.

We overexpressed the recombinant URH1p protein in *Escherichia coli* cells and purified it to homogeneity. During purification, the recombinant protein eluted as a single peak from a size-exclusion chromatography (SEC) column, with a retention volume corresponding to a monomeric species (Figure 2A).

However, we observed the formation of homodimers in samples kept at 4 °C for two weeks, as shown in sedimentation velocity AUC experiments where a monomeric species with a sedimentation coefficient of 2.4 S was predominant and a dimer at 3.5 S was also apparent. Notably, the ratio between monomeric and dimeric URH1p in the sample (~33%) was independent of the protein concentration used in the ultracentrifugation experiment (Figure 2B), indicating a slow rate of the dimer formation process. Dimers could also be observed as a lower mobility band in non-reducing SDS-PAGE that was absent when a reducing agent was used (Figure 2C). We concluded that URH1p is primarily monomeric but can form disulphide-linked dimers through cysteine oxidation (also confirmed by the structural studies; see below). This analysis reveals that active group I NHs can also exist in the monomeric form and that the dimeric or tetrameric quaternary assembly so far observed in this enzyme group is not a strict requirement to support the enzymatic activity.

### 2.2. Analysis of the Substrate Specificity of URH1p

**Figure 3:**
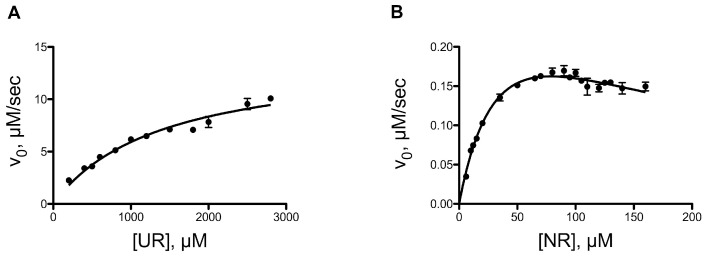
Kinetic analysis of URH1p hydrolysis of ribosides. (**A**) Hydrolysis of uridine follows Michaelis-Menten kinetics in the substrate concentration range tested. (**B**) NR hydrolysis shows a decrease in the initial velocities at high substrate concentration, indicative of substrate inhibition.

To further characterise the substrate specificity and catalytic efficiency of the enzyme, we performed a steady-state kinetic analysis of URH1p at 37 °C (Table 1, Figure 3). Unlike earlier studies, the present kinetic analysis was carried out in phosphate buffer since Tris was shown to interact with several conserved amino acid residues involved in the binding of substrates at the active site of both groups I and II NHs [11,18,35] and is expected to interfere with the determination of the initial velocities.

In our study, URH1p catalysed the hydrolysis of the N-glycosidic bond in the pyrimidine nucleoside uridine with a turnover number of 284 s^−1^, a value that is unparalleled in other NHs so far characterised and much higher than previously measured in Tris-buffered reaction mixtures [15]. Cytidine is hydrolysed much less efficiently, largely due to a lower k_cat_ value (12 s^−1^). The synthetic substrate 5-fluorouridine (FUR) is also converted to products efficiently, with a slower turnover compared to uridine but also a smaller K_M_ value. Under the rapid equilibrium hypothesis, this may indicate a higher affinity of FUR towards the URH1p active site, yet leading to a less productive conformation for the hydrolytic reaction compared to the natural nucleoside uridine. The URH1p-catalysed NR hydrolysis displays a 40-fold lower turnover number, hence implying that the active site of the enzyme achieves a better stabilisation of the transition state for the hydrolysis of uridine compared to that of the pyridine riboside or cytidine. Conversely, the Michaelis’ constant for uridine substrate is 30-fold larger compared to NR. The catalytic efficiencies k_cat_/K_M_ for the two substrates, representing the pseudo-first-order rate constant of the reaction when [S] << K_M_, imply that at physiological, low-micromolar riboside concentrations, the two substrates are converted to ribose and the corresponding nitrogenous base at similar rates, thus allowing URH1p to simultaneously participate in both nucleobase and NAD+ salvage. Notably, NR hydrolysis does not follow simple Michaelis-Menten kinetics, and substrate inhibition was observed with a K_IS_ = 136 µM that is three times the K_M_ value (Figure 2).

### 2.3. Crystal Structure of URH1p, a Monomeric Group I NH Enzyme

To gain further insights into the substrate specificity of the enzyme and the structural features that stabilise the monomeric structure, we determined the crystal structure of recombinant URH1p. The hexagonal crystals appeared after approximately three weeks of incubation of the protein with the precipitant solution, and their diffractions of X-rays were highly anisotropic (Table 2). Scaling of the data with STARANISO allowed the inclusion in the refinement of the high-resolution data that were measured along the c* direction to increase the ratio between observations and parameters in the refinement and improve the electron density maps.

**Figure 4:**
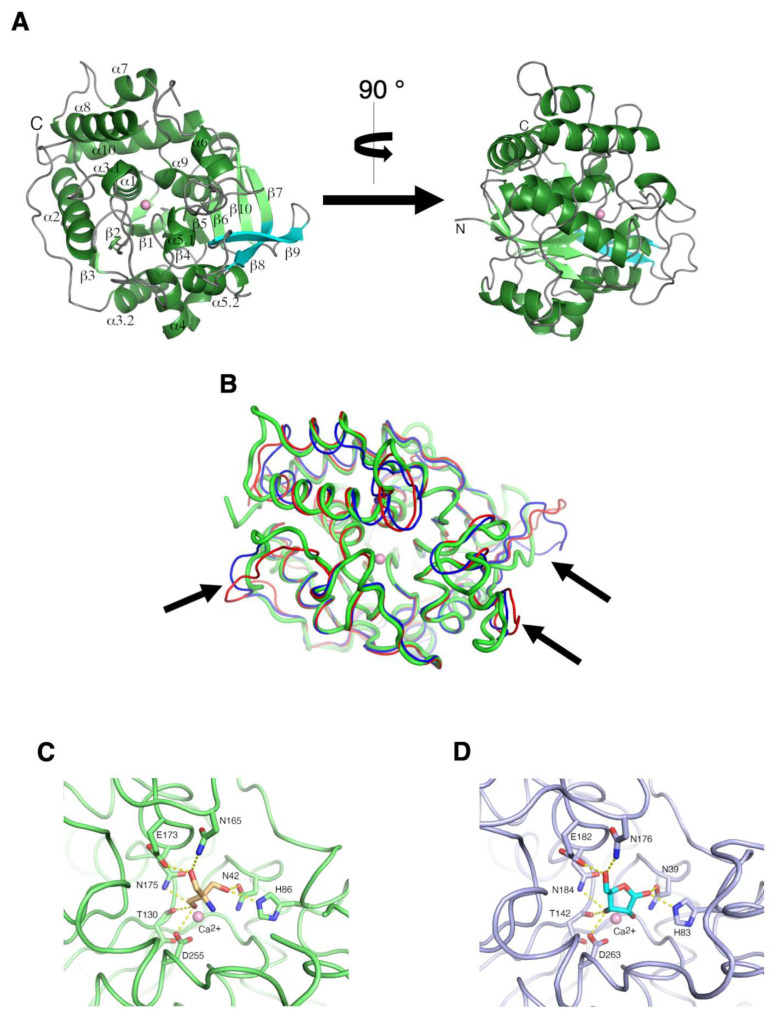
Crystal structure of URH1p from *S. cerevisiae*. (**A**) Ribbon diagram of the overall structure or URH1p. Helices are coloured dark green, strands are light green, and loop regions are grey. The active site Ca^2+^ ion is shown as a pink sphere. The β8 and β9 strands, involved in the oligomerisation of group I NHs, are coloured cyan. (**B**) Superposition of URH1p (green) with the *C. fasciculata* IUNH (red) and *E. coli* RihB (blue), with arrows highlighting the three regions of structural divergence. (**C**) Binding of a Tris molecule from the crystallisation buffer at the URH1p active site. (**D**) Binding of ribose to the *Trichomonas vaginalis* RH (PDB code 8OIA), highlighting that the residues engaged by the Tris molecule in URH1p are involved in the recognition of substrate and product.

The asymmetric unit of the hexagonal crystals contains two URH1p monomers, covalently linked by a disulphide bridge involving residue Cys142 from both chains. The two monomers are very similar in structure (rmsd 0.06 Å for all atoms). Thus, the analysis presented here is carried out using the polypeptide chain termed “A” in the deposited PDB file. The URH1p structure retains the characteristic open (α, β) NH fold [27,30] that is composed of an eight-stranded central mixed β-sheet (β1-β7 and β10) surrounded by the connecting α-helices, a helical domain that completes the active site, and two additional antiparallel β-strands (β8 and β9) extending away from the core sheet (Figure 4A). Among the deposited, experimentally determined structures of NH enzymes, the URH1p structure shows the highest structural homology with the *Crithidia fasciculata* IU-NH (29% sequence identity) in complex with a transition state-like inhibitor (PDB 2MAS) (Figure 4B). A root mean square distance (rmsd) of 1.9 Å is computed over 313 aligned Cα atoms, while this value is reduced to 0.66 Å if computed using 192 structurally homologous Cα atoms with rmsd less than 2.0 Å. Thus, the core structure of URH1p closely resembles the three-dimensional architecture of group I NHs.

The crystal structure of URH1p closely resembles the “closed” conformation observed for the ligand-bound NHs, with an ordered loop that connects strand β3 to helices α3.1 and α3.2 (the so-called “β3-α3 loop”) and a defined conformation of the α8 helix with only its short C-terminal portion (amino acids 242 to 246) flexible, as judged by the weak electron density. Since ligand-free group I NHs typically display high flexibility in the β3-α3 loop and α8 helix [11,22,27], we infer that the contacts with the Tris molecule found in the active site stabilise the observed closed conformation (Figure 4C and Figure S1). Indeed, the side chain imidazole ring of His86, a crucial residue in modulating NH activity [18] and structural dynamics [36], is within hydrogen bonding distance from one hydroxyl of the Tris molecule, and this interaction is likely pivotal for the closed conformation of the β3-α3 loop. Further contacts are observed between the Tris molecule and amino acid residues Asn42, Thr130, Asn165, Glu173, Asn175 and Asp255, whose homologues are involved in substrate binding in other NHs (Figure 4C,D and Figure S2). Hence, the structure reported here exemplifies the conformation that URH1p adopts when in complex conditions with substrates that engage the catalytic residues.

### 2.4. Specific Amino Acid Substitutions Prevent URH1p Oligomerisation

The monomeric structure of URH1p, as evidenced by the chromatographic behaviour, is unprecedented in group I NHs. Group I NHs so far have been characterised as displays of either a homodimeric or homotetrameric quaternary structure [30]. In the latter case, the assembly can be described as a dimer of the dimers, with two different interaction surfaces involved in establishing a structure with 222 (D2) symmetry (Figure S3). In tetrameric NHs, the major interaction surface involves the two antiparallel β-strands β8 and β9, not part of the core sheet, that interact with the same elements from a second protomer. The second minor surface is composed of amino acids from the β3-α3 connecting region and helices α4.1 and α5.2. Despite these amino acid stretches displaying low sequence homology within NHs belonging to group I, the quaternary structure is generally highly conserved [30].

In URH1p, the β8 and β9 strands are more divergent in conformation, and, perhaps most importantly, the amino acid residues exposed at the surface are different in character. The outermost β9 strand is longer compared, for instance, to the one found in the *Crithidia* IU-NH, and both the preceding and following loop regions are packed closer to the URH1p monomer, making them less available for intermolecular interaction. In addition, the optimal matching of hydrophobic or hydrophilic residues between interacting monomers in oligomeric NHs could not be achieved in URH1p. For instance, residue Met274 in the *Crithidia* IU-NH is replaced by Lys296 in URH1p, and upon dimerisation, the latter would be found between the side chains His161, Lys296 and Ile298 of the opposite monomer. The net result of these differences is the inability of URH1p to form stabilising interactions with a second monomer through the region that constitutes the major interaction surface in dimeric and tetrameric NHs.

**Figure 5:**
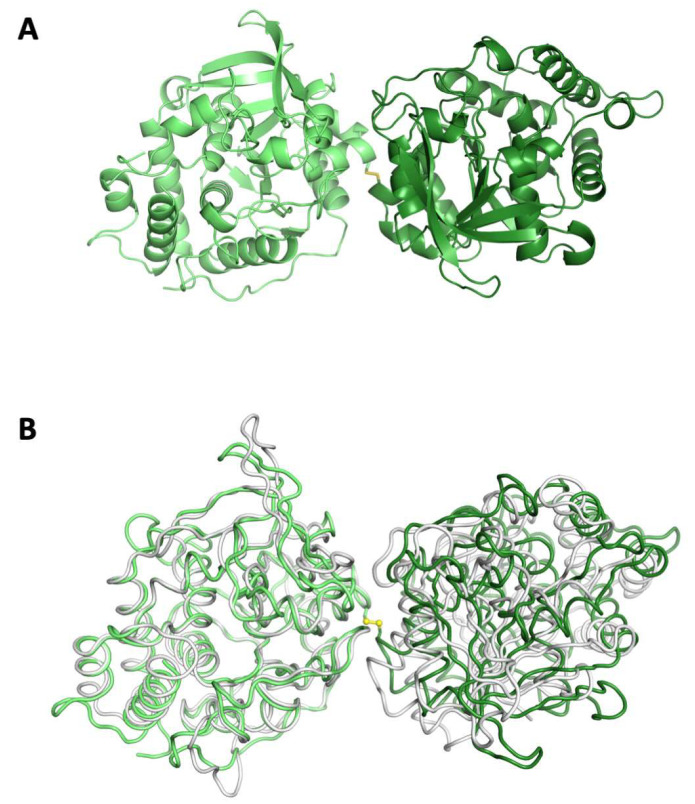
Disulfide-linked dimers in the URH1p crystals. (**A**) Ribbon diagram of the URH1p asymmetric unit content, showing the disulfide linkage between the two independent polypeptides coloured in different shades of green. (**B**) Superposition of the disulfide-linked URH1p polypeptides with the *Crithidia* IUNH dimer formed through minor surface interaction. The superposition was carried out using the atoms of one monomer (left portion of the panel), resulting in a significant displacement of the second subunit.

Instead, the regions that contribute to the second, minor interaction surface in oligomeric NHs are structurally conserved in URH1p (Figure 5A) and are in contact with the second polypeptide found in the crystal asymmetric unit. Nonetheless, the analysis of this interaction surface using PISA computes a solvation energy gain of only −1.1 kcal·mol^−1^ for the intermolecular interaction. The buried surface in this apparent “dimer” is the smallest so far reported for group I NHs (490 Å^2^ in URH1p, with a range between 674 and 807 Å^2^ in bona fide oligomeric NHs), with only Lys76 in close contact with the side chains of both Asp181 and Asp287 at optimal distance for charge-charge interactions. Additionally, the guanidinium group of Arg141 from monomer A approaches the indole ring of Trp75 of monomer B, albeit with an orientation that is not ideal for cation-π interactions (Figure S3). Such a URH1p “dimer” can be superimposed with a rmsd of 2.8 Å for 492 Cα atoms with the corresponding pair from the IU-NH from *C. fasciculata* (chains A and C, PDB code 2MAS). However, if only one monomer is used in the superposition, the calculated rmsd for 296 matching atoms of the counter-lateral monomers is 6.8 Å, while the same analysis performed between the tetrameric *Crithidia* IU-NH and the *E. coli* RihB NH enzymes (PDB code 1Q8F) returns a computed rmsd of 4.3 Å over 298 Cα. Hence, although the two URH1p chains in the asymmetric unit interact via the minor dimerisation surface of canonical NHs, the resulting assembly is quite divergent from what is observed in close structural homologues (Figure 5B). Making only marginal non-covalent interactions between the two protein monomers in the asymmetric unit, it is thus evident that the URH1p dimer found in the crystal is principally caused by an adventitious disulphide bridge between Cys142 residues, not representing a physiologically relevant quaternary arrangement. Intriguingly, the monomeric state of URH1p demonstrates that NH oligomerisation is under active, selective pressure, possibly modulated by evolutionarily diversified species-specific metabolic requirements.

### 2.5. Modelling of the URH1p-Substrate Interactions

**Figure 6:**
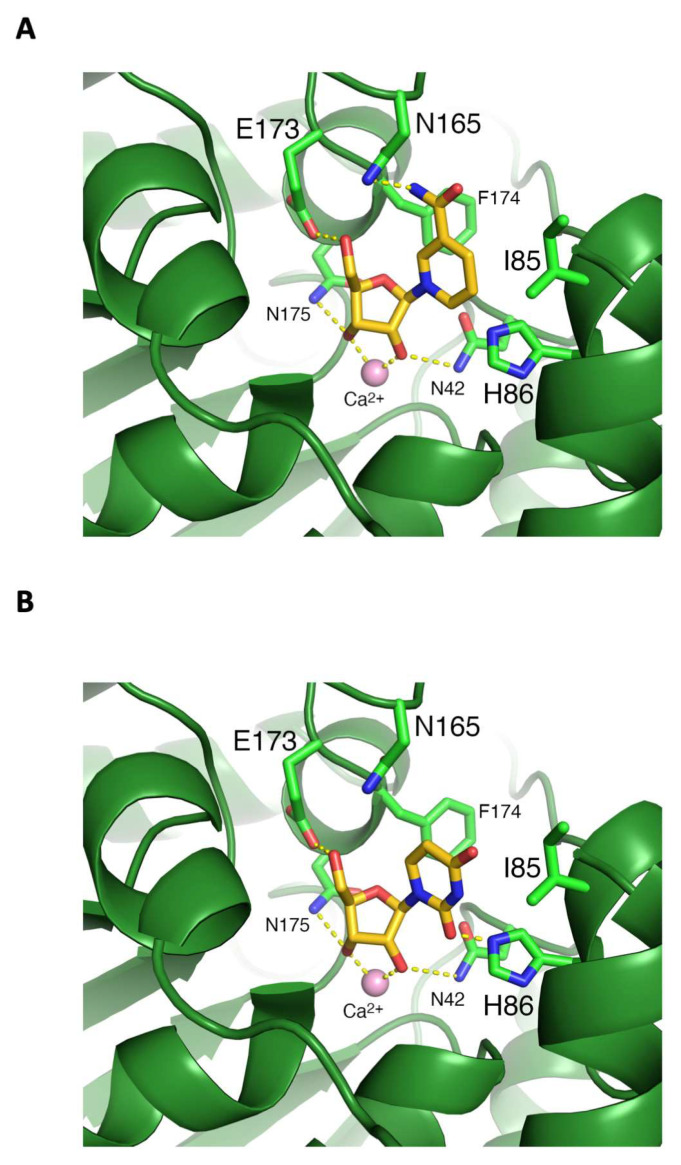
Modelling of the URH1p-substrate interactions. (**A**) The NR molecule positioned in the active site can interact with residue Asn165. (**B**) Uridine can be accommodated with the O2 carbonyl in close proximity to His86, which can stabilise the negative charge developing at the transition state.

The fast turnover of substrates by URH1p does not allow the determination of the structure of enzyme-substrate complexes by cocrystallisation or soaking followed by cryotrapping, as was possible, for instance, for the *E. coli* RihB in complex with inosine [35]. Thus, to gain further insights into the active site residues that may interact with the principal URH1p substrates, we modelled the URH1p-uridine and URH1p-NR complexes using the structure of the *C. fasciculata* IU-NH bound to the transition state-like inhibitor 4-aminophenyl iminoribitol as a template. First, we assumed an axial conformation of the base and a C4′-endo ribosyl conformation, as determined in both the template complex structure and in the E. coli RihB-inosine complex. With these constraints, we performed a minimisation in vacuo of the structure of the URH1p substrates uridine and NR and superimposed the resulting riboside conformation to the iminoribitol-based inhibitor in the *Crithidia* isozyme. The modelled NR substrate fits the URH1p active site with the nicotinamide ring positioned between the side chains of Ile85 and Phe174. The carboxyamide group closely approaches the amide side chain of residue Asn165 (Figure 6A) with the potential to establish a bidentate hydrogen bond. The opposite orientation of the nicotinamide would clash with the side chain of residues Ile85 and His86, part of the β3-α3 loop. When uridine is modelled in the active site of URH1p, the O2 oxygen of the pyrimidine base closely approaches the imidazole side chain of residue His86, an amino acid residue whose mutation affects the turnover number of uridine for the enterobacterial RihB NH (Figure 6B).

### 2.6. Thermal Stability of URH1p

**Figure 7:**
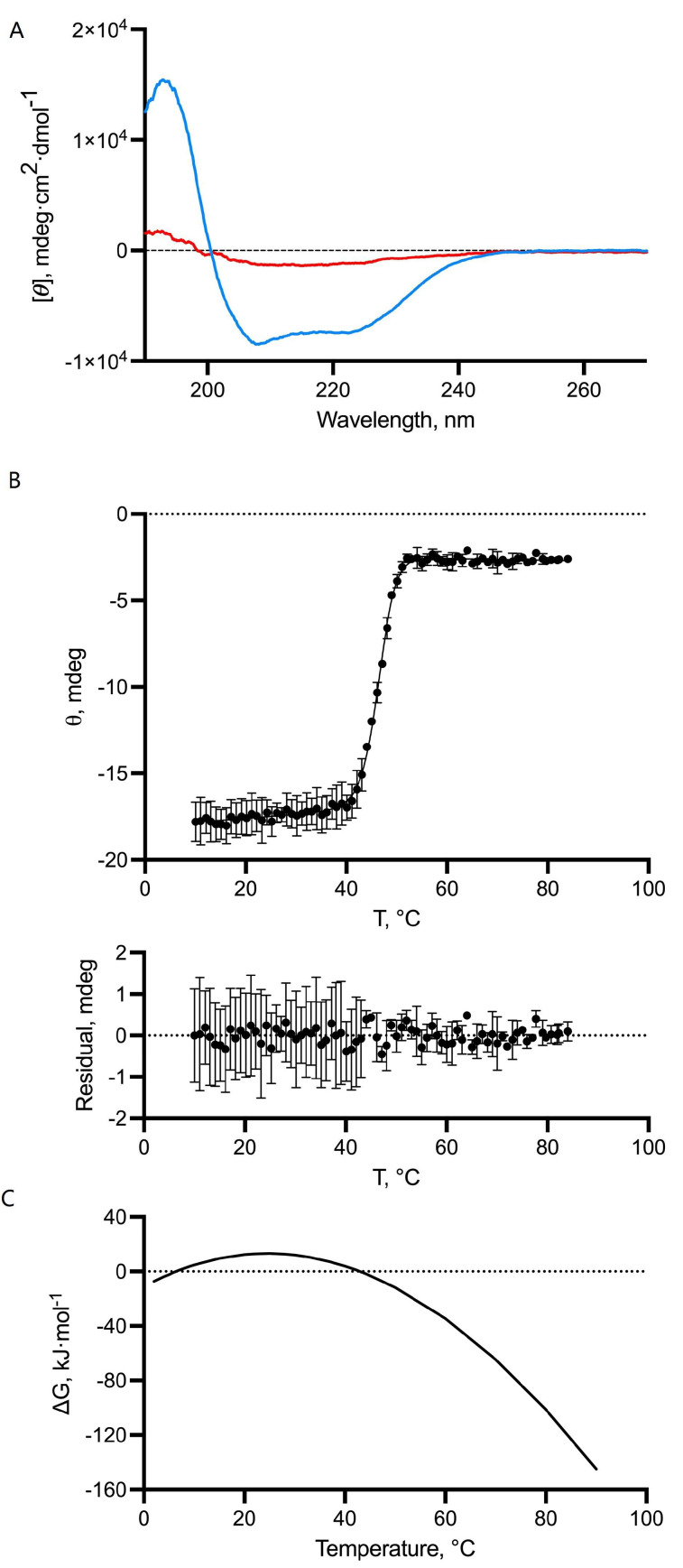
Analysis of the thermal stability of URH1. (**A**) Circular dichroism spectra of purified, recombinant URH1p at 25 °C (blue) and 85 °C (red). (**B**) The thermal unfolding curve of URH1p shows a sharp transition between the two states. The curve was fitted as described in the text. The bottom panel show the residuals after the fitting. In both panels, error bars represent standard deviations from the mean (*n* = 3). (**C**) Calculated unfolding ΔG values for URH1p as a function of temperature based on the experimentally determined ΔH and ΔS values.

To explore the possibility of taking advantage of the activity of URH1p on the fluorinated nucleoside 5-fluorouridine in cancer therapy, we wished to assess the stability of URH1p as a function of temperature. To this end, we first monitored the thermally-induced unfolding of URH1p through the variation in CD signal (Figure 7A) at increasing temperatures [38]. The sigmoidal curve obtained is characteristic of a single transition with a T_m_ = 47.0 ± 0.5 °C, without evidence of intermediate unfolding states (Figure 7B). At this temperature, the unfolding enthalpy ΔH^0^ amounts to 540 ± 40 kJ·mol^−1^, corresponding to 1.6 kJ·mol^−1^·residue^−1^, and the entropy variation ΔS^0^ equals to 1.7 kJ·mol^−1^·K^−1^. The optimal fit of the CD data was obtained considering a positive variation in specific heat at constant pressure (ΔC_p_) of 23.5 ± 2.4 kJ·mol^−1^·K^−1^, which is indicative of a predominant contribution of apolar group hydration upon unfolding [39]. Then, using the values of the thermodynamic parameters obtained at the T_m_ as a reference value, an inverted U-shaped curve of ΔG for the folded-unfolded transition as a function of temperature with a maximum at T = 24.5 °C is obtained, indicating a thermal stability range for URH1p between 5 and 44 °C (Figure 7C). It should be noted that the values obtained refer to the isolated protein that could be further stabilised in vivo by ligands or intermolecular interactions.

## 3. Discussion

The landscape of ribosides that are substrates of group I NHs has widened considerably in recent years. Seminal work on the yeast URH1p demonstrated that the enzyme is involved in the nicotinamide salvage pathway by catalysing the N-ribosidic bond hydrolysis in the pyridine riboside NR. URH1p activity, together with nucleoside and methylthioadenosine phosphorylases, is crucial for NR-dependent NAD^+^ synthesis that ultimately sustains the biological outcomes deriving from the activation of the protein deacetylase Sir2. Sirtuin activity extended the lifespan of yeast cells similarly to what was observed under conditions of calorie restriction [13]. The here presented kinetic analysis of URH1p differs from what was previously reported [15], likely due to the different conditions used in the enzymatic reaction. Previous studies on the catalytic properties of URH1p showed an 8-fold higher k_cat_/K_M_ for the NR substrate compared to uridine due to a lower K_M_ value for the former compound. Our results agree with most catalytic parameters for the two substrates, except for a 14-fold greater k_cat_ value for uridine that we attribute to the different experimental conditions used, mainly the inhibitory effect of Tris that was used in the earlier analysis. Indeed, we found that Tris binds to URH1p, engaging several catalytic site residues, and this may justify the discrepancies in the kinetic parameters previously obtained using this compound as a buffering agent. Our analysis indicates that URH1p has a 2-fold greater catalytic efficiency (k_cat_/K*_M_*) towards NR compared to the best natural nucleoside substrate, uridine. NR hydrolysis does not follow classic Michaelis-Menten kinetics, and substrate inhibition was apparent with a K*_IS_* = 136 µM that is three times the K_M_ value. Nucleosides are present in living cells at micromolar concentrations since they are neutral and are substrates of equilibrative nucleoside transporters and may thus diffuse outside the cell. To the best of our knowledge, the range of concentrations of nucleosides or NR in yeast cells has not been determined, and the only value reported is NR concentration in human plasma, which is ~10 µM [40]. Thus, the low K_M_ value for NR allows the enzyme to act efficiently at the presumably low cytoplasmic concentration. The relatively similar catalytic efficiencies here reported suggest that uridine and NR compete for URH1p-mediated hydrolysis.

It is well established that group I NHs catalyse the hydrolysis of ribosides through ground state destabilisation, distorting the ribose geometry through enzyme-substrate contacts while stabilising the negative charge that develops in the nitrogenous base [41,42]. Specifically, NHs interact with the ribosyl moiety of the substrates through coordination of the active site Ca^2+^ ion by the O2′ and O3′ hydroxyls, together with a network of hydrogen bonds to highly-conserved protein residues [43]. The resulting C4′-endo puckering of the ribose represents a high-energy conformation that approaches the oxonium ion geometry of the NH-catalysed transition state as determined by multiple kinetic isotope effects [41]. The negative charge developing at the nitrogenous base is neutralised through proton transfer, and two conserved histidine residues are involved in this process in group I NHs [17,18,24]. Differences in turnover number for cytidine and uridine are apparent in URH1p, in line with those previously shown for the other NH enzymes RihA and RihB [18,22], and consistent with diversities in the enzyme-mediated stabilisation of the negative charge in the pyrimidine ring. While pyrimidine nucleoside hydrolysis apparently relies on the presence of only one histidine residue, the turnover of purine nucleosides requires a more complex network of amino acids, including two tyrosine side chains, to shuttle a proton to the N7 atom [35]. The NR molecule contains a pyridinium ion, and thus, its hydrolysis is expected to proceed primarily through the ribosyl distortion since it does not require protonation of the leaving group [11]. The model of NR bound to URH1p suggests that this substrate may favourably interact with residue Asn165 and Phe174, aiding the attainment of the axial conformation of the β-N-ribosidic bond to reach the transition state. The hydrolysis of pyrimidine nucleosides catalysed by URH1p apparently proceeds along an at least partly distinct reaction path. Uridine has a much faster turnover compared to NR. Thus, it can reach the transition state structure more efficiently through specific interactions with the enzyme, which include the stabilisation of the uracil base product. Perhaps surprisingly, the presence of the electron-withdrawing fluorine atom at position 5 in FUR decreases the rate of the URH1p-catalyzed hydrolysis, advocating that the stabilisation of the negative charge in the leaving base may not be the rate-limiting process in the URH1p-catalyzed nucleoside hydrolysis. This result differs from what is shown for the *E. coli* RihB pyrimidine-preferring NH, where the k_cat_ value for different 5-halouridines correlated with the electron-withdrawing properties of the substituent [18]. Thus, despite the shared architecture of the active site, the hydrolysis of pyrimidine nucleosides catalysed by NHs is finely tuned by isozyme-specific enzyme-substrate contacts.

URH1p is the first group I NH enzyme that displays a monomeric structure, and all homologues so far characterised are stable as either dimers or tetramers [30]. While in group II NHs that include the purine-specific isozymes, the quaternary structure is crucial for activity [44], the role of oligomerisation in group I in modulating the hydrolytic activity has been debated. Although the effects may be isozyme-dependent and not necessarily be generalised, our findings show that the individual URH1p polypeptide is stable and catalyses the hydrolytic reaction with efficiencies that are comparable to or surpass what is demonstrated for other group I homologues. Thus, the formation of dimers or tetramers is not a strict requirement for attaining activity in group I NH enzymes. Yet, most of the isozymes in the group so far studied in protozoa and bacteria are tetrameric, while two enzymes characterised by plants are dimeric. Oligomerisation may thus be a factor in enhancing protein stability or may facilitate protein-protein interactions with cellular partners. The interactome of NHs, as evidenced by high-throughput studies, is heterogeneous and not immediate in its interpretation, including proteins from diverse, unrelated pathways [45,46]. More detailed, specific studies are required to clarify the interplay between NH structure and other pathways.

**Scheme 2:**
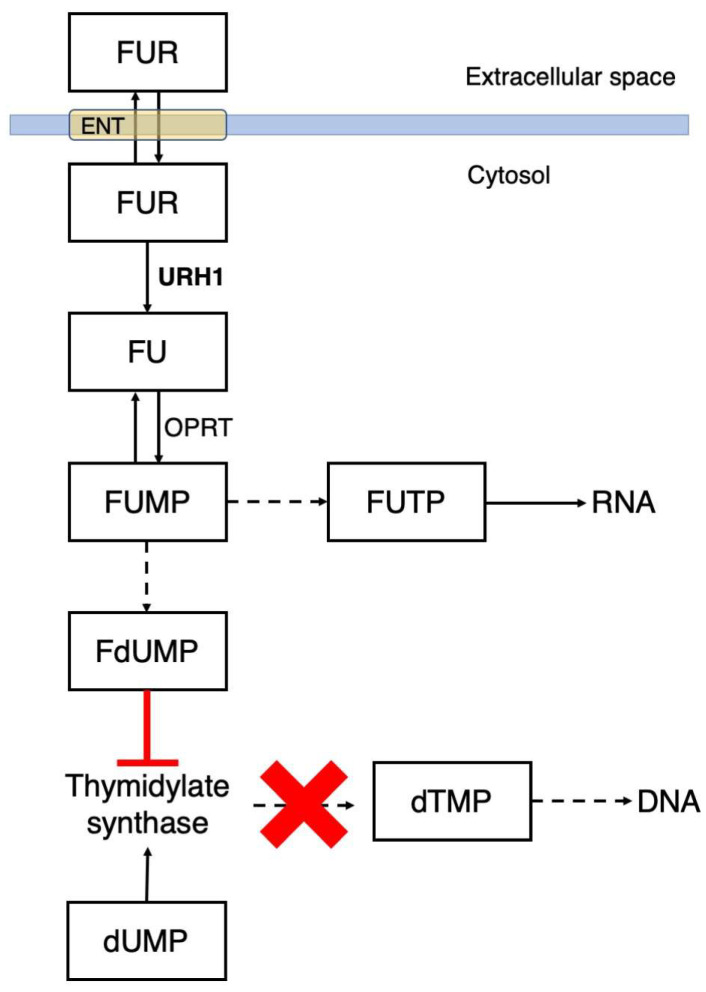
Mechanism of activation and action of the chemotherapeutic agent FU and its precursors in cancer cells. While other mechanisms may also contribute to the cytotoxicity of fluorinated pyrimidines, the interference with RNA folding and thymidylate synthase activity are the major targets.

The compound 5-fluouracil and its derivatives, such as capecitabine, have been long used as the cornerstone of chemotherapeutic intervention against several tumours, including colorectal cancer [47]. The fluorinated base can be incorporated in nucleotides via a phosphoribosyl transferase-mediated reaction, and these can have a cytotoxic effect by disrupting RNA structure as well as ultimately being converted to the 5-fluorodeoxyuridylate that covalently inhibits thymidylate synthase [48] (Scheme 2). The toxic fluorinated nucleotides are also transferred to neighbouring cells, leading to a bystander effect that amplifies the therapeutic efficacy of the drug [49]. Although this mechanism is highly appealing, as it efficiently targets rapidly dividing cells, the pyrimidine catabolic pathway leads to 5-fluoruracil inactivation primarily through the dihydropyrimidine dehydrogenase enzymatic activity. The hydrolytic activity of URH1p on the synthetic nucleoside FUR may be exploited to partially circumvent this problem. Indeed, FUR displays higher bioavailability but poor conversion to the mononucleotide in mammalian cells. The ectopic expression of URH1p in tumour cells in combination with FUR administration may thus be used as novel tools for gene-directed enzyme-prodrug activation therapy approaches. While the efficacy of this approach in cells remains to be fully demonstrated, the characterisation of NHs that hydrolyse FUR with high catalytic efficiency could provide relevant tools for therapeutic development. URH1p has a threefold greater k_cat_/K*_M_* for FUR hydrolysis compared to the *E. coli* RihB NH [18] and would be thus able to rapidly convert low nucleoside concentrations inside cells to the free base and achieve a therapeutic effect more efficiently.

The size of the *urh1* gene (1020 base pairs) does not pose difficulties for its transfer via non-integrating viral vectors, and its expression may be optimised through organ-selective promoters. As an alternative strategy, the compact structure of monomeric URH1p can facilitate its encapsulation in lipid- or polymer-based delivery systems that can be equipped with tumour-selective receptors to achieve selectivity [33,34]. Finally, the demonstrated stability of URH1p at physiological temperatures makes it amenable to achieve sustained prodrug activation. Taken together, the present results on the FUR-activating activity of the URH1p enzyme warrant further validating studies in tumoral cell lines and model systems as an alternative tool for a tumour-directed GDEPT approach. Moreover, the structural and enzymatic characterisation of URH1p shows this enzyme may outperform kinetically other NHs, making this protein suitable for the development of matched pro-drugs to move the effectiveness of GDEPT beyond FUR.

## 4. Materials and Methods

### 4.1. Cloning Expression and Purification

The urh1 gene was amplified using polymerase chain reaction (PCR) from yeast genomic DNA using the gene-specific forward and reverse primers 5′-CACCGCTAGCACTGTTAGTAAAATACCCATATGG-3′ and 5′-TTACTCGAGTTATCCAATCGTTGACATTTTG-3′, including the (underlined) *NheI* and *XhoI* restriction sites, respectively. The gel-purified PCR product was cloned between the same sites of the pET28b plasmid vector for protein expression. A single colony of BL21(DE3) cells transformed with the construct was used to inoculate a starter culture that was grown for 16 h at 37 °C in Luria-Bertani (LB) medium. The culture was diluted 1:100 in LB and grown at 37 °C under vigorous shaking to A_600_ = 0.6. Cells were harvested by centrifugation at 4000× *g*, washed and resuspended in fresh LB medium and protein expression was induced by the addition of 0.5 mM isopropyl β-D-thioglucopyranoside. Protein expression was carried out for 16 h at 18 °C. Cells were harvested by centrifugation and resuspended in 1/10th of the culture volume in a buffer composed of 20 mM Tris pH 8, 50 mM NaCl and a cOmplete EDTA-free protease inhibitor cocktail (Sigma-Aldrich, Milano, Italy). Cells kept on the ice were disrupted by sonication using a Bandelin signifier. The soluble fraction was separated by centrifugation at 20,000× g and applied to a HisTrap affinity column coupled to an AktaPure FPLC system. The protein was eluted using a linear imidazole gradient. The affinity-purified URH1p was dialysed against 20 mM Tris pH 8.4, 150 mM NaCl, 2.5 mM CaCl_2_ and then incubated with thrombin (Merck, Milano, Italy) in a 1:200 (*w*/*w*) ratio for 16 h at 4 °C. Quantitative removal of the affinity tag was confirmed by Western blot analysis using a peroxidase-conjugated anti-His_6_ antibody. The reaction mixture was loaded onto a Superdex 200 16/60 size exclusion chromatography column and eluted isocratically in 20 mM Hepes pH 7.4 and 150 mM NaCl. Protein concentration was measured by measuring the absorbance at 280 nm and using a value of ε_280_ = 48,360 M^−1^·cm^−1^ as calculated from the amino acid sequence.

### 4.2. Analytical Ultracentrifugation

Sedimentation velocity (SV) analytical ultracentrifugation data were collected using a Beckmann/Coulter XL-I analytical ultracentrifuge (Beckman Coulter, Cassina de’ Pecchi (MI), Italy) equipped with a double-sector centrepiece with sapphire windows. Radial spectra were acquired in absorbance mode (280 nm) at five different protein concentrations (0.1, 0.2, 0.4 and 0.6 mg·mL^−1^, corresponding to 2.6, 5.2, 10.4 and 15.6 µM) at 45,000 rpm (163,004 g). The experimental curves were deconvoluted to c(S) distributions using the software SEDFIT v16-1c [50].

### 4.3. Circular Dichroism

Circular dichroism (CD) spectra were recorded in the 180–270 nm range using a Jasco J-815 spectropolarimeter (Jasco Europe, Cremella (LC), Italy) equipped with a Peltier cell for temperature control and using a 1 mm cuvette. The protein was extensively dialysed against a buffer composed of 50 mM sodium-potassium phosphate at pH 7.4 and 150 mM NaF prior to collection of the spectra and adjusted to a final concentration of 5 µM. Wavelength scans were performed at a rate of 20 nm·min^−1^. To monitor the thermal unfolding of URH1p, the variation in CD signal was recorded at three different wavelengths (209, 220, and 222 nm) while the temperature varied at a rate of 1 °C/min. The CD signals were converted to molar ellipticity using a calculated molar mass of the recombinant protein of 37,960 Da. The experimental ellipticities as a function of temperature were fitted using different models, considering either constant or variable specific heat at constant pressure (C_p_) and accounting for a linear CD signal drift before and after the transition. The optimal fit was assessed from the comparison between the error statistics and the distribution of residuals. The equations used for the fitting are reported as Supplementary Material.

### 4.4. Steady-State Kinetic Analysis

Kinetic measurements were performed on an UltroSpec 2100 UV spectrophotometer (GE Healthcare, Milano, Italy), monitoring the variation in absorbance at the appropriate wavelength for each substrate [11,19] upon conversion to the nucleobase. The variation in molar absorption (Δε) between nucleoside and nucleobase and wavelengths used were uridine, −2.0 µM^−1^·cm^−1^ at 280 nm; inosine, −0.82 µM^−1^·cm^−1^ at 280 nm; 5-fluorouridine, −1.86 µM^−1^·cm^−1^ at 269 nm; nicotinamide riboside −3.1 µM^−1^·cm^−1^ at 266 nm. Reactions were carried out in 20 mM sodium-potassium phosphate buffer at pH 7, and enzyme concentrations used in the assays were 50 nM for uridine, cytidine and FUR substrates and 20 nM for NR. Initial velocities were determined from the slope of the linear portion of the progress curve where the conversion of the substrate to the product was <10%. Kinetic parameters for each substrate were obtained from the fitting of the initial velocities measured at eight different substrate concentrations (*n* > 3) to the Michaelis-Menten equation using GraphPad Prism v.5. For nicotinamide riboside, the data were fitted to the equation for substrate inhibition:

(1) $$v_{0}=\frac{V_{max}[S]}{K_{M}+[S](1+\frac{\left[S\right]}{K_{IS}})}$$

### 4.5. Crystallisation and Structural Determination

Single crystals of URH1p were obtained using the sitting drop vapour diffusion method. Equal volumes of URH1p (10 mg/mL) and a precipitant solution composed of 100 mM Tris·HCl (pH 8.5), 800 mM LiCl and 32% PEG 4000 were mixed using an Oryx8 robot (Douglas Instruments, East Garston, UK) in MRC 2 well plates (Hampton). Crystals grew at 18 °C to maximum dimensions of 50 µm. Crystals were harvested using nylon loops, transferred into a stabilising solution composed of the precipitant solution supplemented with 15% PEG 400, rapidly dipped into liquid N_2_ and kept at 100 K for data collection.

X-ray diffraction data were collected from a single crystal at beamline ID23-2 of the European Synchrotron Radiation Facility (ESRF, Grenoble, France) using the oscillation method and a Dectris Eiger X 9M detector. Data were indexed, integrated, scaled, and reduced to unique reflections using the programs XDS [51], AIMLESS [52], and STARANISO, as implemented in the software autoProc [53]. A CC_1/2_ > 0.5 cut-off was applied for the inclusion of high-resolution reflections in the measured dataset [52,54]. Initial phases were obtained using the molecular replacement technique as implemented in MOLREP [55], using the E. coli RihB monomer structure as a search model (PDB code 1Q8F) after removal of ions, ligands and water molecules. The structure was rebuilt manually into σ_A_-weighted (2 mF_o_-DF_c_, ɸ_c_) and (mF_o_-DF_c_, ɸ_c_) electron density maps in Coot [56], followed by maximum likelihood refinement in Refmac5 with non-crystallographic symmetry restraints applied [57]. Difference density maps revealed the presence of the expected Ca^2+^ ion at the active site, as well as one molecule of Tris from the crystallisation buffer. Individual B-factors were refined, and one set of TLS parameters for each independent chain in the asymmetric unit was used to model anisotropy. Forty solvent molecules were added in the later stages of refinement in spherical residual density peaks within hydrogen bonding distance from protein residues. The maximum resolution of the usable diffraction data was established using the paired refinement technique, ensuring that the inclusion of further data did not improve R_free_ or the R_free_-R_crys_ difference [54]. The geometry of the model was monitored at every cycle using Molprobity [58]. The refined model contains amino acids 2 to 340 of the mature URH1p protein, except for the residues in the loop region 242–246 at the end of helix α8 that had weak electron density associated and were thus excluded from each monomer. Data collection and refinement statistics are presented in Table 2. Structural analysis and rmsd calculations were performed using PyMol (http://www.pymol.org, accessed on 23 June 2024). Intermolecular contacts were analysed using PISA [59]. Structure factors and refined coordinates have been deposited with the Protein Data Bank (PDB), accession code 8RIH (https://doi.org/10.2210/pdb8rih/pdb).

### 4.6. Modelling of Substrate-Enzyme Contacts

The coordinates of the competitive, transition-state-like inhibitor pAPIR (2-(4-aminophenyl)-5-hydroxymethyl-pyrrole-3,4-diol) were extracted from the coordinates of the complex with the *C. fasciculata* IU-NH (PDB code 2MAS). The inhibitor structure was used as a template to obtain uridine and NR models by manually adding and replacing atoms using the Avogadro software [60]. The structures of the riboside substrates were minimised in vacuo, applying constraints on the ribose ring puckering and N-glycosidic bond orientation and using the steepest descent algorithm with the UFF force field. The energy-minimized ligands were placed manually in the active site using the software Coot based on the position of the iminoribitol ring of the pAPIR molecule in complex with the *C. fasciculata* IU-NH [43] after superposition with URH1p.

## 5. Conclusions

The specificity, catalytic efficiencies and regulation of the URH1p enzyme from *S. cerevisiae* allow a role as a central player in both the uptake pathway of NR and nucleobase salvage. URH1p is a unique example within the so far characterised riboside hydrolases by virtue of its monomeric structure. The stability of the enzyme at physiological temperatures and its efficient hydrolysis of the synthetic nucleoside FUR make it an attractive tool for a prodrug activation therapeutic approach against solid tumours.

## Figures and Tables

**Table 1:** Steady-state kinetic parameters of URH1p.

	k_cat_ (s^−1^)	K_M_ (µM)	k_cat_/K_M_ (×10^5^ M^−1^·s^−1^)	K_IS_ (µM)
Uridine	284 ± 14	1400 ± 143	2.0 ± 0.2	-
Nicotinamide riboside	17.5 ± 1.5	45 ± 6	3.9 ± 0.6	136 ± 23
Cytidine	12 ± 1.7	1020 ± 80	0.12 ± 0.02	-
5-fluorouridine	162 ± 10	467 ± 19	3.5 ± 0.4	-
Inosine	<0.001	n.d.	n.d	-

**Table 2:** Crystallographic analysis of URH1p.

Data Collection			
Beamline	ID23-2 ESRF		
Wavelength (Å)	0.87313		
Unit cell parameters (Å)	a = b = 140.284 c = 81.343 α = β = 90° γ = 120°		
Space group	P6_3_		
Diffraction limits (Å)	a* = b* = 3.73 c* = 2.41		
Resolution range (Å)	121.49–2.60	121.49–9.13	2.89–2.6
R_pim_	0.167	0.025	0.988
Total reflections	177,292	8483	7714
Unique reflections	13,763	687	688
Multiplicity	12.9	12.3	11.2
Mean I/σ(I)	5.9	16.9	1.8
Completeness (spherical, %)	49.0	99.6	9.3
Completeness (ellipsoidal, %)	94.1	99.6	81.8
CC_1/2_	0.981	0.996	0.570
**Refinement statistics**			
Resolution range (Å)	121.49–2.74		
N. of reflections (free set)	12,828 (714)		
R_crys_	0.188		
R_free_	0.225		
rmsd bonds (Å)	0.003		
rmsd angles (°)	0.907		
N. of atoms	5,280		
**B-factors (Å^2^)**			
Protein	11.0		
Solvent	21.4		
Tris	22.7		
**Geometry analysis**			
Ramachandran plot statistics (% favoured, outliers)	97.1, 0.3		
Molprobity score	1.69		
Clashscore	6.18		

## Data Availability

The structure factors used for the crystal structure determination and the coordinates of the refined URH1p model have been deposited with the Protein Data Bank (http://www.rcsb.org/pdb accessed on 23 June 2024) under accession code 8RIH.

## Supplementary Materials

The following supporting information can be downloaded at: https://www.mdpi.com/article/10.3390/ijms25137032/s1.
