# Supplementary material for: Structure-Function Insights into the Dual Role in Nucleobase and Nicotinamide Metabolism and a Possible Use in Cancer Gene Therapy of the URH1p Riboside Hydrolase
Source: Int J Mol Sci. 2024 Jun 27;25(13):7032. doi: 10.3390/ijms25137032 (PMC11241417; doi:10.3390/ijms25137032)
Supplement: Supplementary file 1 [file ijms-25-07032-s001.zip › ijms-3032749-supplementary.pdf]

Structure-function insights into the dual role in nucleobase and nicotinamide metabolism and a possible use in cancer gene therapy of the URH1p riboside hydrolase

Alejandra Angela Carriles, Laura Muzzolini, Claudia Minici, Paola Tornaghi, Marco Patrone and Massimo Degano

Supplementary material

Figure S1. Electron density of the Tris molecule bound to URH1p
Figure S2. Comparison of the binding or ribose to the Trichomonas riboside hydrolase with the binding of Tris to URH1p
Figure S3. Intermolecular contacts between the URH1p independent subunits in the crystal
Supplementary text. Equations used for the fitting of the thermal unfolding CD data

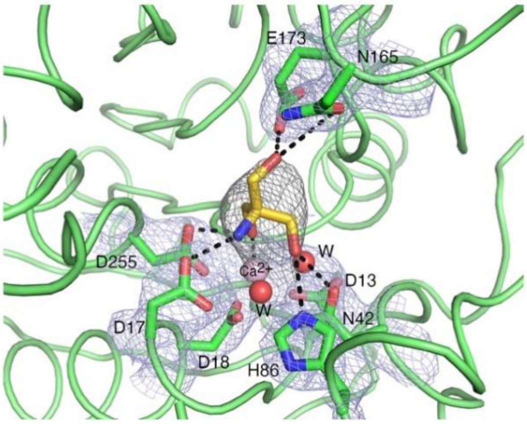

Figure S1. Electron density of the Tris molecule bound to URH1p. The (F_o-F_c, ϕ_c) electron density of the Tris molecule (shown at 3.0 σ contour level) was calculated after omitting the ligand molecule, applying a random shift to a final rmsd of 0.3 Å to the atoms, and re-refined with Refmac5. The ($2F_o$-F_c, ϕ_c) density (contoured at 1.2 σ) is shown for the protein residues.

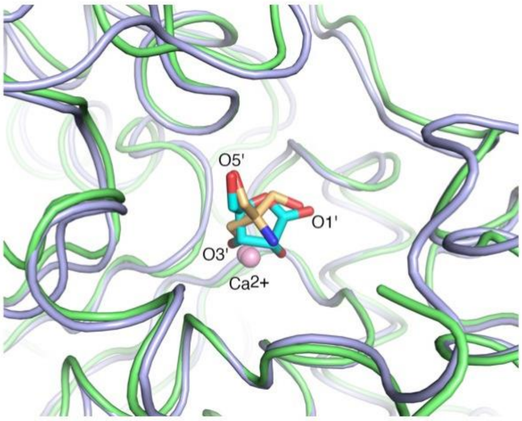

Figure S2. Comparison of the binding or ribose to the *Trichomonas vaginalis* riboside hydrolase with the binding of Tris to URH1p. The two protein structures were superimposed with Pymol using the Cα carbons. The three hydroxyls of the URH1p-bound Tris molecule occupy the positions of the O1′, O3′ and O5′ hydroxyls of the ribose molecule in the parasite's enzyme (PDB code 8OIA).

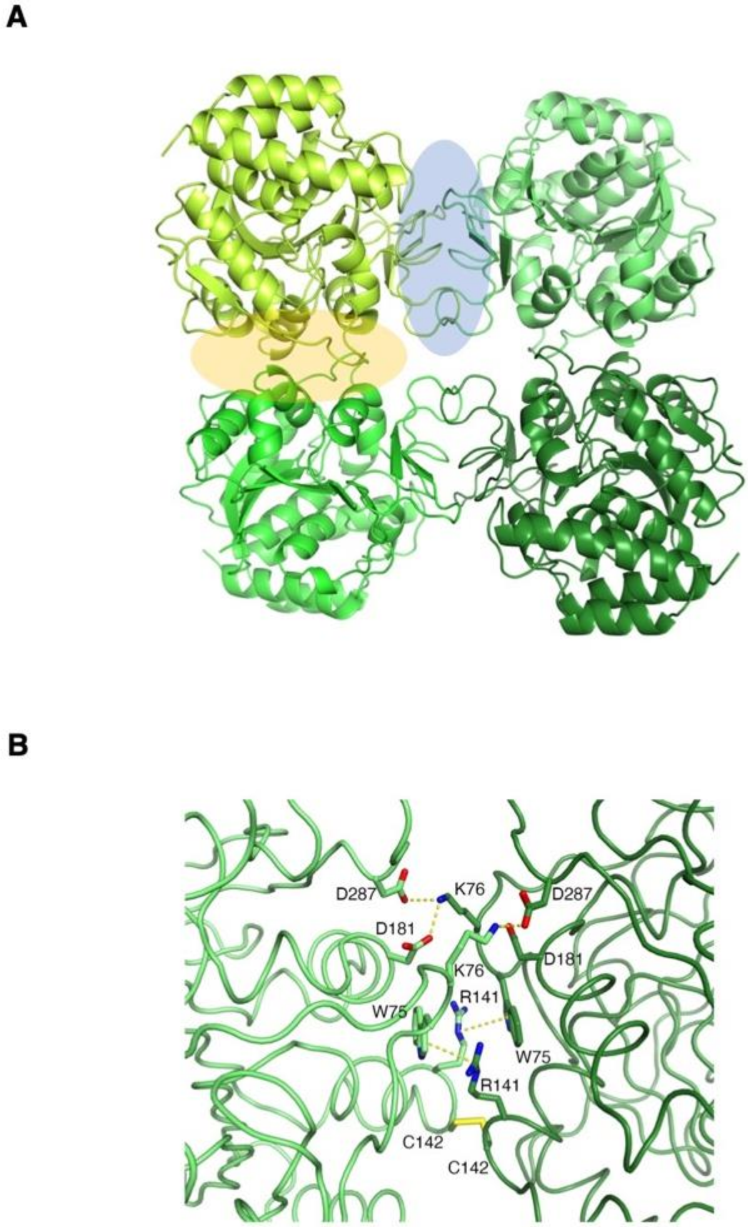

Figure S3. Intermolecular contacts between the URH1p independent subunits in the crystal. (**A**) Cartoon representation of the epitomic group I NH, the IUNH from C. fasciculata (PDB code 2MAS). The four subunits are colored in different shades of green. The most extensive intermolecular interaction surface (“major” surface) is highlighted by a light blue oval, the minor in yellow. (**B**) Intermolecular interactions between the two URH1p subunits in the asymmetric unit.

Supplementary text.
Equations used for the fitting of the URH1p unfolding following the variation in CD spectra.

Considering the unfolding process:

$$F \rightleftarrows U$$

The associated equilibrium constant is:

$$K = \frac{[U]}{[F]}$$

At any temperature T, at the equilibrium the fraction of unfolded protein is:

$$\alpha = \frac{[U]}{[F]+[U]} = \frac{K}{1+K}$$

At a given temperature T:

$$K = e^{-\frac{\Delta G}{RT}} = e^{-\frac{\Delta H}{RT}+\frac{\Delta S}{R}}$$

At the melting temperature, α=0.5, thus K=1. Hence, at this temperature:

$$K = 1 \;\; \Rightarrow \; \Delta S = \frac{\Delta H(T_M)}{T_M}$$

At a generic temperature, T:

$$K = e^{\frac{\Delta H(T_M)}{R}\left(\frac{1}{T_M}-\frac{1}{T}\right)} = e^{\frac{\Delta H}{RT}\left(\frac{T}{T_M}-1\right)}$$

If C_P is not constant, then:

$$\Delta H = \Delta H(T_M) + \Delta C_p(T - T_M)$$
$$\Delta S = \Delta S(T_M) + \Delta C_p ln\left(\frac{T}{T_M}\right)$$

Hence, the more general equation for K is:

$$K = e^{\frac{\Delta H(T_M)\cdot\left(\frac{T_M}{T}-1\right)-\Delta C_p\left[T_M - T + T ln\left(\frac{T}{T_M}\right)\right]}{RT}}$$

The ellipticity at any temperature point is given by:

$$\theta_T = (\theta_f + D_1 T) + \left[(\theta_u + D_2 T) - (\theta_f + D_1 T)\right]\alpha$$

Where D_1 and D_2 are the slopes of the drift in signal before and after transition, if present. Parameters to be fit are θ_f, θ_u, T_M, $\Delta H(T_M)$, D_1, D_2, and ΔC_p.
Once the values are obtained, the thermodynamic parameters can be computed at any temperature using:

$$\Delta H = \Delta H(T_M) + \Delta C_p(T - T_M)$$
$$\Delta S = \Delta S(T_M) + \Delta C_p ln\left(\frac{T}{T_M}\right)$$
$$\Delta G = \Delta H(T_M) - T\Delta S(T_M) + \Delta C_p\left[(T - T_M) - Tln\left(\frac{T}{T_M}\right)\right]$$

Taking the first derivative of ΔG, the maximum stability is at:

$$T = T_M e^{-\frac{\Delta S(T_M)}{\Delta C_p}}$$
